# An Optically-Transparent Aptamer-Based Detection System for Colon Cancer Applications Using Gold Nanoparticles Electrodeposited on Indium Tin Oxide

**DOI:** 10.3390/s16071071

**Published:** 2016-07-12

**Authors:** Mojgan Ahmadzadeh-Raji, Ebrahim Ghafar-Zadeh, Ghasem Amoabediny

**Affiliations:** 1 Biologically Inspired Sensors and Actuators ( BioSA) Laboratory, Department of Electrical Engineering and Computer Science, Lassonde School of Engineering, York University, Toronto, ON M3J1P3, Canada; m_ahmadzadeh@ut.ac.ir; 2 Department of Life Science Engineering, Faculty of New Sciences & Technologies, University of Tehran, Tehran 14395-1561, Iran; 3 Department of Biotechnology and Pharmacy Engineering, Faculty of Chemical Engineering, University of Tehran, Tehran 4563-11155, Iran; 4 Research Center of New Technologies in Life Science Engineering, University of Tehran, Tehran 1417-963891, Iran

**Keywords:** apta-DS, Gold Nanoparticle (GNP), indium tin oxide (ITO), cyclic voltammetry (CV), colon cancer, chronopotentiometry (CP)

## Abstract

In this paper, a label-free aptamer based detection system (apta-DS) was investigated for detecting colon cancer cells. For this purpose, we employed an aptamer specific to colon cancer cells like HCT116 expressing carcinoembryonic antigen (CEA) on their surfaces. Capture aptamers were covalently immobilized on the surface of gold nanoparticles (GNPs) through self-assembly monolayer of 11-mercaptoundecanoic acid (11-MUA) activated with EDC (1-Ethyl-3-[3-dimethylaminopropyl]carbodiimide)/N-hydroxysuccinimide (NHS). The cyclic voltammetry (CV) and chronopotentiometry (CP) methods were used for electrodeposition of GNPs on the surface of indium tin oxide (ITO). In this work, the CV method was also used to demonstrate the conjugation of GNPs and aptamers and identify the cancer cell capturing events. Additionally, Field Emission Scanning Electron Microscopy (FE-SEM) confirmed the deposition of GNPs on ITO and the immobilization of aptamer on the apta-DS. The electrodeposited GNPs played the role of nanoprobes for cancer cell targeting without losing the optical transparency of the ITO substrate. A conventional optical microscope also verified the detection of captured cancer cells. Based on this study’s results relying on electrochemical and optical microscopic methods, the proposed apta-DS is reliable and high sensitive with a LOD equal to 6 cell/mL for colon cancer detection.

## 1. Introduction

Recent advances of nano-biotechnologies and their applications for life sciences have received significant attention for early detection of a variety of cancerous diseases such as colon cancer [1,2]. Colon cancer is the leading cause of cancer death in the world [3,4]. Despite the great recent advances, millions of people are suffering from this disease while receiving harsh treatments such as chemotherapy [5,6]. Therefore, development of reliable cost-effective devices, such as aptamer based detection system (apta-DS), for early detection of colon cancer metastasis remains a key challenge. For this purpose, circulating tumor cells (CTCs) not only can be considered as an important biomarker for early detection purposes, but they are also the best candidate for monitoring treatment progression [7,8].

**Figure 1:**
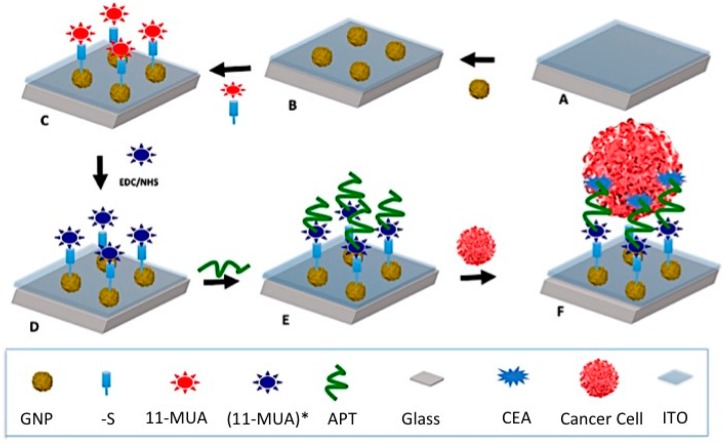
Illustration of the apta-DS fabrication process: (**A**) ITO coated glass; (**B**) Electrodeposited GNPs on ITO substrate (Working electrode); (**C**) Deposition of 11-MUA on GNPs; (**D**) Activation of 11-MUA using EDC/NHS; (**E**) Bonding of aptamer and activated 11-MUA [or so called (11-MUA) *] and (**F**) cancer cell attachment on aptamer.

To date, many papers have reported the advantages of sensors for the diagnosis of prostate, breast and colon cancers [9,10,11,12]. Among the few papers detailing the use of apta-DSs for colon cancer diagnosis, Sheng et al. reported a microfluidic device incorporated with a large number of micro-pillars used for isolating HCT 116 cancer cells from a blood sample [13]. The biotinylated aptamer was immobilized on these micro-pillars coated with avidin. In these techniques, the detection of colon cancer relies on quantitative fluorescence microscopy. Other researchers reported the detection of cancer cells by directly immobilizing MUC1 aptamers on the surface of gold or carbon nano-spheres (CNSs) using electrochemical measurement techniques [14,15]. In another effort, our research team previously reported the immobilization of an aptamer on a gold electrode via a strong covalent bonding between the aptamer and the 11-mercapto-undecanoic acid (11-MUA) absorbed as a self-assemble monolayer on the surface of gold electrode [16]. The CV experimental results demonstrated the high sensitivity of our previously reported apta-DS, with a limit of detection (LOD) equal to 7 cell/mL. In this paper, we investigated this approach further, developing an apta-DS consisting of an optically transparent ITO coated with GNPs using an electrodeposition technique [17,18,19]. In this work, the GNPs decorating the ITO substrate enhanced the attachment of cancer cells on the apta-DS without losing optical transparency. Therefore, the proposed optically transparent apta-DS can take the advantages of both optical microscopy and electrochemical sensing techniques. On other words, the optical technique can be used as further verification of the sensor’s performance. For this reason, the proposed apta-DS offers a reliable technique for the detection of cancer cells. Figure 1 depicts the fabrication steps to form the apta-DS described in this paper. These steps include electrodeposition of GNPs, immobilization of 11-MUA on GNPs, the activation of COOH group in 11-MUA and KCHA10a modified truncated aptamer with a (CH_2_)_6_ linker bonded to 11-MUA [16,20]. The aptamers target the whole cell which carcinoembryonic antigen (CEA) express on the surface of colon cancer cells to trap these cells.

As aforementioned, the electrodeposition technique is used to modify the surface of the ITO electrode using GNPs as colloidal particles. Such particles suspended in a liquid medium can migrate under the influence of an electric field and deposit on the surface of electrode. This electrodeposition technique is widely used to coat the surface of ITO with GNPs for an accelerated electron transfer rate. According to current literature, potassium dicyanoaurate KAu(CN)_2_ or chloroauric acid HAuCl_4_ is widely used in electrodeposition to grow GNPs on the surface of glass coated with thin ITO films using CV. The electrodeposition process can be controlled using various parameters including the voltage range (V_R_), number of cycles (N_C_) and scan rate (S_R_). For instance, by selecting V_R_ = −0.4 to −1.3 V, N_C_ = 20, S_R_ = 50 mVs^−1^, the diameter size of GNPs is about 20 nm [17,18,19]. In this paper, we report a new protocol to deposit the previously synthesized GNPs on the surface of ITO using CV so as to develop an apta-DS dedicated to early detection of colon cancer.

## 2. Materials and Methods

In this section, we describe the materials and methods including the preparation of GNPs, electrodeposition of GNPs on ITO, immobilization of the synthesized aptamer on GNPs, sample preparation and sample test on apta-DS.

### 2.1. GNPs Preparation and Characterization

In this work, GNPs were prepared in five steps. In the first step, 1 mmol/L of HAuCl_4_·3H_2_O was dissolved in 250 mL water (ultra-purification Milli-Q system, Millipore Inc., Darmstadt, Germany), then stirred with a magnet and heated up to 100 °C. Thereafter, 25 mL of 38.8 mmol/L trisodium citrate was added to the solution and stirred again for 15 min until the color of the solution become Cerise [21].

The size and size distribution of GNPs are analyzed using dynamic light scattering (DLS) and ultraviolet-visible (UV-Vis) spectroscopy techniques, which are the most commonly used methods in this field of research [22,23]. DLS is used to determine the size distribution profile of nanoparticles in suspension [24]. On the other hand, UV-Vis spectroscopy technique is used to measure the absorption/reflectance spectroscopy of the nanoparticle suspension in the UV and visible spectral regions with a wavelength (λ) range from 200–800 nm. The wavelength of maximum absorbance (λ_max_) is a characteristic value that varies by size, shape, and concentration of GNPs. UV spectrophotometry (Model Ce 1010, Cecil, UK), DLS (Brookhaven Company, New York, NY, USA) and zeta potential (Brookhaven Company, USA) techniques were used for the optical characterization, size distribution and the surface charge of generated particles in this process. After electrodeposition on ITO, field emission scanning electron microscopy (FE-SEM) (MIRA3 TESCAN, Razi Foundation, Iran) was used to evaluate the morphology and size distribution of the GNPs coated on the ITO surface.

### 2.2. Electrodeposition of GNPs on ITO

The synthesized GNPs were electrodeposited on the ITO using chronopotentiometry (CP) and cyclic voltammetry. The CP and CV processes were performed at 25 °C in the solution of synthesized GNPs remaining from part A (GNPs Preparation and Characterization) while the nitrogen gas was purged in the solution. In this electrochemical cell, a Pt electrode and an Ag/AgCl electrode are used as counter (CE) and reference (RE) respectively. Also a glass substrate coated with ITO is used as a working electrode (WE). The resistivity of WE electrode consisting of an ITO thin film coated onto a glass substrate (Sigma Aldrich, London, UK) is equal to 30–60 Ω/Square. The dimensions of WE are about 0.5 and 2.5 cm. These WE, CE and RE electrodes were connected to Ivium potentiostat (IVUIM Technologies model IVIUMSTAT.XR, Fernandina Beach, FL, USA) for electrochemical measurements and analysis using Ivium software (Version 2.42.5 Ivium soft). The excitation voltage of CP varied from 2.5 to 0.2 over a 60 s time interval. This potential-time curve reveals the oxidation and reduction of GNPs on the surface of ITO. After the CP, a CV process was performed with a scan rate of 50 mVs^−1^ and a voltage range from −0.2 to 0.8 V for 90 cycles. As a result of this process, the color of the ITO electrode coated with GNPs was inverted to red. The remaining solution was removed and the electrochemical cell was washed using double distilled water. Then another CV process was performed with the same scan rate and voltage range for 5 cycles using a PBS solution containing 1 mM concentration of K_3_[Fe(CN)_6_] adjusted at a pH equal to 7.4, while the nitrogen gas was purged in the solution at a temperature of 25 °C. It is noteworthy that the ITO on glass substrate decorated with GNPs is used as the working electrode (WE) and Ag-AgCl and Pt electrode were used as the reference electrode (RE) and counter electrode (CE) in the cyclic voltammetry experiments described in Section 2.2 and Section 2.3. The characterization of electrodeposited GNPs is also performed using an image processing technique. In this paper, an open-platform scientific image analysis software, Image J, was used to measure the size distribution of GNPs that were observed in FE-SEM images.

### 2.3. Immobilization of Capture Aptamer on GNPs

The GNPs-decorated ITO electrode was soaked in a buffer containing 20 mM of 11-MUA (Sigma Aldrich, London, UK) for one day. This buffer is an ethanol/H_2_O solution 3:1 (*v*/*v*). The electrode was thereafter washed with ethanol and H_2_O, and dried with nitrogen gas. The electrode was then immersed in phosphate buffer saline (PBS) containing 5 mM N-hydroxysuccinimide (NHS) and 2 mM N-ethyl-N-(3-diethylaminopropyl) carbodiimide (EDC) (Sigma Aldrich, London, UK). This buffer was adjusted to a pH of 7.4. Thereafter, the electrode was soaked in a Tris-HCl buffer (pH = 7.6 and ionic strength I = 0.14) containing 0.4 µM of modified aptamer KCHA10a with (CH_2_)_6_ as a linker which is truncated of aptamer KCHA10 [16,20]. The electrodes were ultimately dipped in a deionized water containing 1% Bovine Serum Albumin (BSA) for 30 min and then in a Tris-HCl buffer (Gibco^®^, Thermo Fisher Scientific, London, UK) for 10 min. This final rinse was applied to remove unbounded BSA. It is noteworthy that SELEX is employed to search for the aptamer DNA sequence. KCHA10a is extracted through this process and functionalized by adding a linker and a label, namely amino-C6 at the 3′ end and fluorescein isothiocyanate (FITC) at the 5′ end respectively [25]. This modified aptamer, called FITC-DNA Aptamer RAJI2-HP (Tag Copenhagen Inc., Frederiksberg, Denmark) is 5-FITC-ACGCAGCAGGGGAGGCGAGAGCGCACAATAACGAT-GGTTGGGACCCAACTGTTTGGACA-Amino-C6-3. It is noteworthy that FITC is a fluorescence label that is not used this paper.

### 2.4. Cell Preparation and Attachment

Two types of epithelial cancer cell lines, namely HCT 116 (NCBI code: C570) and HEp-2 (Pasteur Institute Inc., Tehran, Iran), were used as target and control cells respectively. The cells were cultured in a medium (RPMI, Bioeideh Company, Tehran, Iran) containing 300 mg/L of L-glutamine, 100 μg/mL Penicillin, 100 IU/mL Streptomycin and 2.5 μg/mL Amphotericin B. The required fetal bovine serum (Nano Zist Arrayeh Company) for cell culture contains various factors such as epidermal growth and adhesion factors in addition to antitrypsin activity in order to promote cell proliferation and attachment in adherent flasks (JET BIOFIL, Guangzhou, China). The incubator (Sanyo MCO-17AI, LabX, Tokyo, Japan) was set at 37 °C with a carbon dioxide concentration equal to 5%. The passage of confluent control cells were performed with trypsinizing the cells using 0.25% w/v trypsin plus 0.53 mM ethylenediaminetetraacetic acid (EDTA) solution [9]. The passage of confluent target cells was different from control cells. Instead of trypsin, a cell scraper was used to detach HCT 116 from the flask. This simple process prevents the CEA proteins expressed on the surface of target cells from trypsin damage. After centrifuging, a PBS buffer was used to dissolve the cancer cells. In the last step, the cancer and control cells were introduced on the proposed apta-DS individually. These experiments were repeated using different concentrations (6, 12 and 25 cells/mL) as demonstrated and discussed in Section 3.

### 2.5. Cell Fixation

After introducing the control and colon cancer cells, the apta-DS were kept at room temperature for 20 min. The cells were fixed on the apta-DS using 2.5% of glutaraldehyde (Sigma Aldrich, London, UK) for 30–45 min at 4 °C. Then the apta-DSs were treated with sodium cacodylate buffer (Sigma Aldrich, London, UK) for 10–15 min. After this step, the apta-DSs were immersed in 1% osmium tetroxide solution for 20 to 30 min at room temperature. Thereafter, the apta-DSs were washed with sodium cacodylate and dehydration occurred in various concentrations of 25%, 50%, 70%, 90% and 100% ethanol (Merck, QC, Canada) and dried at room temperature. The latter process was repeated three times.

## 3. Results

In this section, the GNPs synthesis and colon cancer detection results are demonstrated and discussed.

### 3.1. Nanoparticle Characterization: Before and after Electro-Deposition

**Figure 2:**
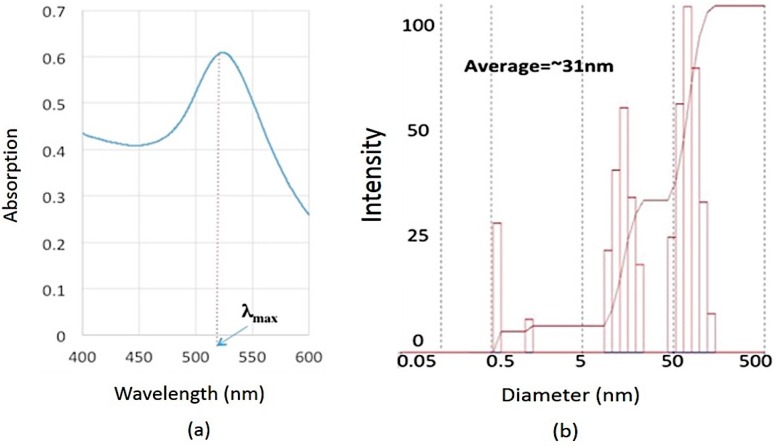
GNPs characterization results using (**a**) UV-Vis absorption spectroscopy; (**b**) DLS technique.

The spherical GNPs demonstrated one single band in the visible region with a maximum wavelength in the range of 520–550 nm. In this work, as seen Figure 2a, UV-Vis spectroscopy results showed that the synthesized GNPs have an absorption maximum at 527 nm wavelength, which confirmed the nanoscale size of GNPs. The DLS also determined that the average size of GNPs was ~31.9 nm. Furthermore, using DLS, the size distribution of GNPs was obtained as seen Figure 2b. This figure shows the synthesis of polydisperse GNPs. Despite this fact, the synthesized GNPs are successfully used in this project. Indeed, each GNP is used as a substrate to immobilize aptamer. Therefore, the size homogeneity of GNPs is not considered as a key factor in developing the proposed system.

**Figure 3:**
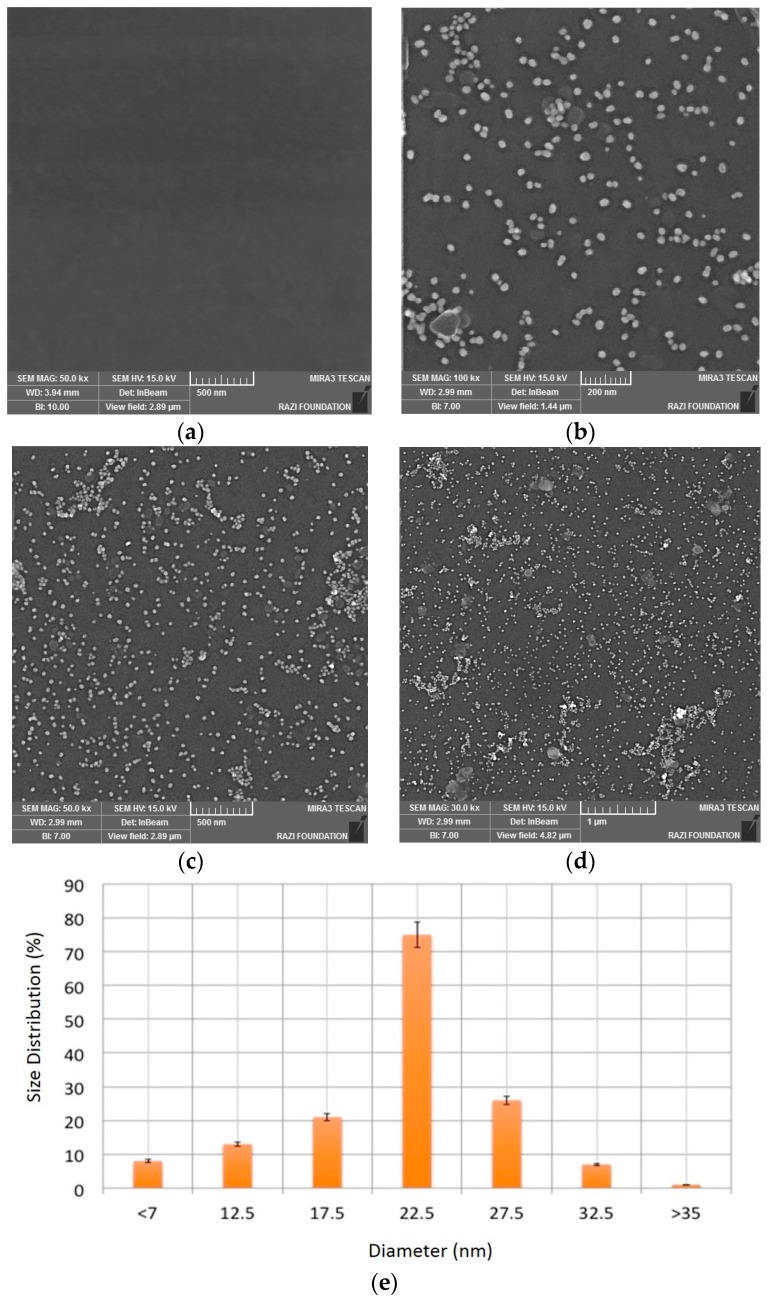
GNPs characterization results using FE-SEM: (**a**) Bare ITO and GNPs-coated ITO with (**b**) 200 nm; (**c**) 500 nm and (**d**) 1000 nm scale bars; and (**e**) an image processing software-Image J. All analyses were repeated for three times.

The zeta potential (ZP) of GNPs synthesized with trisodium citrate was another key parameter to determine the surface charge of nanoparticles in colloidal solution. This surface charge is crucial to prevent the aggregation of GNPs. In this work, ZP = −42.29 mV. A field emission scanning electron microscope (*FE-SEM Model MIRA3 TESCAN, Razi Foundation*) was used to confirm the attachment of GNPs on ITO surface. As seen in Figure 3b–d, the spherical nanoparticles of nearly 25 nm in diameter with a narrow size distribution were electrodeposited on ITO surface. Figure 3a shows the FE-SEM images of a bare ITO. This image is used as a reference/control image.

Figure 3b,c confirm the electro-deposition of GNPs on ITO electrodes. In the small surface area, Figure 3b shows the uniformity of distributed GNPs, however in the larger surface areas, the distribution of GNPs are non-uniform, as seen in Figure 3c,d. These figures (Figure 3c,d) show the deposition of GNPs with higher density that resulted in non-uniform GNPs on ITO. An image processing software-Image J. was used to measure the nanoparticle size and distribution as seen in Figure 3e. As seen in this figure, the average size of nanoparticles is about 22.5 nm. By assuming that the resolution of SEM technique is about 1 nm and using the aforementioned image processing technique, the error in measuring the size of each nanoparticle is about 1/22.5 = ~4.4%. Based on these experimental results, the electrodeposition technique was successfully used to decorate GNPs on ITO substrate. The electrodeposited GNPs allow for the immobilization of aptamers for cancer cell detection as described in the next section. It is noteworthy that using Image J, FE-SEM image (Figure 3b) is considered as the main image to characterize the GNPs deposited on the ITO substrate. The image processing results show the deposition of monodisperse in ITO. However, using Figure 3d, the characterization results may show more than one clusters of GNPs. As aforementioned, still such polydisperse GNPs are useful for the development of apta-DS.

### 3.2. Aptamer Immobilization

**Figure 4:**
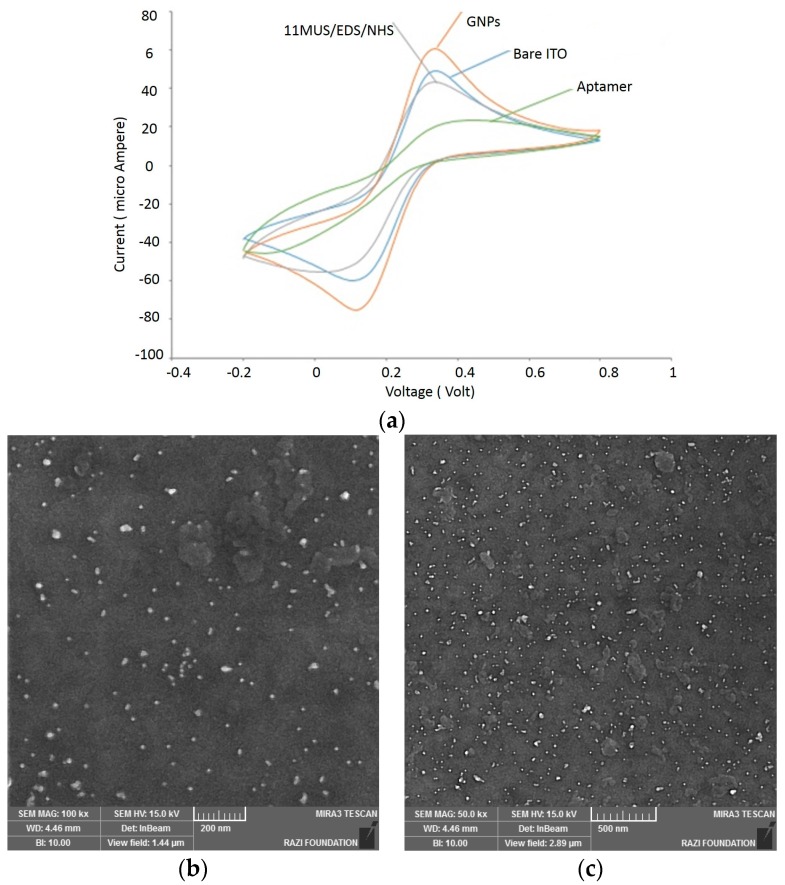
Characterization of apta-DSs (**a**) layer by layer CV characterization of apta-DS consisting of (bare ITO, deposition of GNPs on ITO, deposition of 11MUA/EDC/NHS on GNPs, and covalently bonding of aptamer on functionalized GNPs (11MUA/EDC/NHS/APT/BSA); FESEM microscopy in two different scales (**b**) 200 nm and (**c**) 500 nm scale bars. Electrode coated with 11MUA/EDC/NHS/Aptamer/BSA.

Electrochemical experiments were performed using Ivium potentiostat in a phosphate buffered saline (PBS) containing K_3_Fe (CN)_6_ 1 mM at pH = 7.4 vs. Ag/AgCl. The voltage varies between −0.2 to 0.8 V with a scan rate of 50 mVs^−1^. Figure 4a shows the CV curves related to multiple steps of immobilizing capture aptamers on ITO electrodes. These steps include the bare ITO preparation, deposition of GNPs on ITO, deposition of 11 MUA/EDC/NHS on GNPs and bonding between aptamer/BSA and 11 MUA. The change of current voltammogram in each step showed that the deposition/bonding process in each step is performed properly (Figure 4a). After GNPs deposition, the current oxidation (Ipa) and reduction (Ipc) peaks showed an increase (Figure 4a). This increase was due to the enhancement of electron transfer and conductivity of electrode surface. On the other hand, the deposition of 11 MUA/EDC/NHS on the GNPs change the electrode surface area and consequently it change the Ipa and Ipc peaks.

The electron conductivity of 11-MUA varies depending on the redox probe used during experiments [26], however the activated 11-MUA/EDS/NHS is not charged any more as the reaction of EDS and NHS with 11-MUA results in a stable amide bond [27]. Therefore, the deposition of 11-MUA/EDS/NHS can also be considered as an insulating layer on GNPs. This is why the current associated with the deposition of 11MUA/EDS/NHs is decreased as seen in Figure 4a.

Furthermore, the bonding between the aptamer/BSA and 11 MUA led to a decrease in both Ipa and Ipc currents. The Ipa/Ipc current reduction was due to the decrease of electrode surface area coated by BSA. Figure 4b,c shows the FE-SEM images of apta-DS after forming the linker between the aptamer and ITO surface, and after immobilizing the aptamer.

### 3.3. Cancer Cell Detection

**Figure 5:**
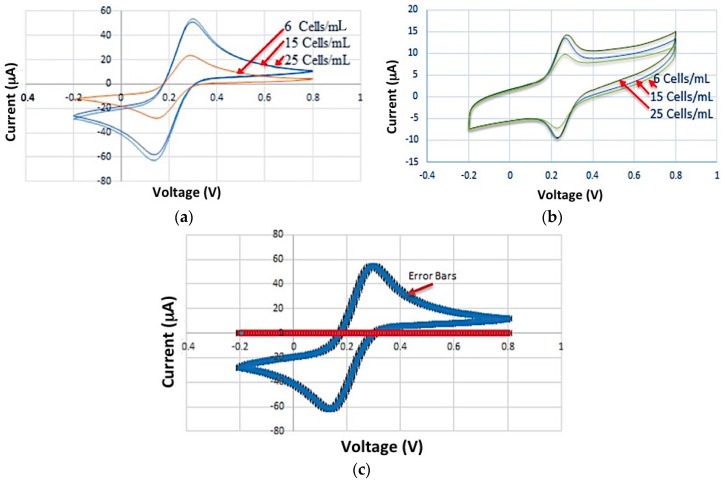
Cyclic voltammetry results related to (**a**) the test of apta-DS exposed to HCT116 cells at various concentrations of 6, 12 and 25 cells/mL; (**b**) the test of apta-DS exposed to HEp-2 cells at various concentrations of 6, 12 and 25 cells/mL and (**c**) the correspondence of cancer and control cells on the same curve. Each experiment related to CV curves was repeated more than three times.

The HCT 116 and HEp-2 cells with different concentrations were introduced to aptamer-coated nanoparticles. Figure 5a,b shows the CV results after the attachment of control and target cells, respectively. As seen in Figure 5a, the introduction of control cells (HEp-2) with different concentrations resulted in the same CV curves. The presence of target cells (HCT 116) with different concentrations can significantly change the current of electrochemical measurement system as seen in Figure 5b. It is noteworthy that the required time for the cells to reach the aptamer is in the range of a few seconds. Therefore the required time elapses after injection of sample in order to perform CV and observe the measurement results is in the same range of a few minutes.

**Figure 6:**
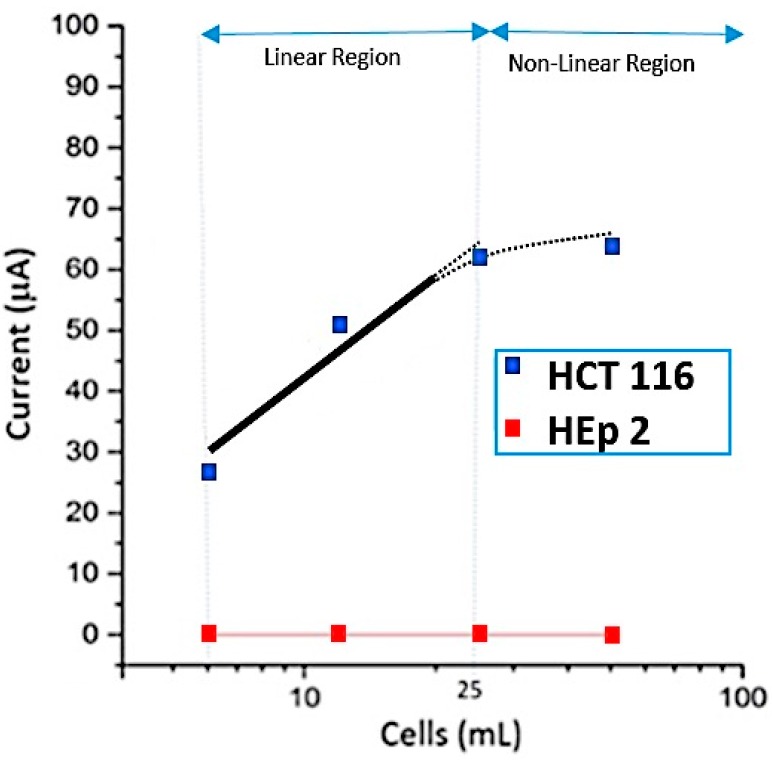
Calibration curve for cancer and control cells. Each experiment related to CV curves was repeated more than three times.

**Figure 7:**
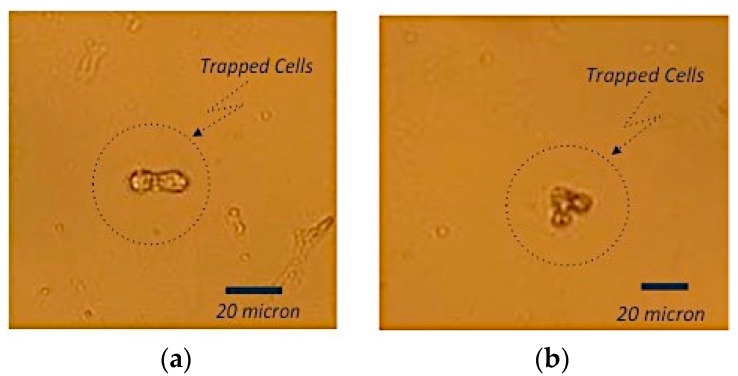
Optical microscopic characterization results: optical microscopic image of apta-DS after introducing target cancer cells with (**a**) two and (**b**) three HCT 116 cells trapped by apta-DS. The scale bar under each SEM image shows that the actual size of HCT 116 is around 10 μm diameter.

As shown in Figure 6, there is a linear relation with a pearson’s r = 0.92 between the output current of electrochemical measurement system versus the number of cells per mL from 1 to around 25 cells/mL. As seen in Figure 6, this relation is not linear when the concentration of cells is more than 25 cells/mL. Based on these electrochemical experiments, the sensitivity of apta-DS using CV measurement technique was not better than 6 cells/mL. Furthermore, the CEA-negative HEp-2 cell line that was used as control and the target cancer cells were introduced to the apta-DS. Based on these experiments, the control cells did not attach on the surface of apta-DS. This is in agreement with the optical microscopic (Olympus model BX50, Tokyo, Japan) images shown in Figure 7.

Thanks to the optical transparency of the proposed apta-DS, the trapped cells by aptemers can be visualized using optical microscope. As seen in Figure 7, the optical microscopic result confirmed the attachment of HCT 116 cells on the apta-DS. Since the non-target cells were easily removed during the rinsing process, the proposed apta-DS consequently offers a reliable and accurate cancer detection technique using optical microscopes available in all biological laboratories.

As seen in Figure 6a, the increase of target cell concentration will increase the output of the proposed apta-DS. Indeed, the presence of target cells in proximity of aptamers immobilized on GNPs increases the probability of binding between the aptamer and target cells. As the result of this binding process, the unfold aptamers become folded and the aptamer conformation rearrangement increases the conductivity. The change of conductivity can be due to the partially desorption of aptamer from the surface of the GNPs due to its conformation. However as seen in Figure 7, still the target cells are trapped by aptamers immobilized on the surface of apta-DS. As the result of this binding process, the oxidation (and the absolute value of reduction) current is increased. The related CV curve is placed in between two curves associated with bare GNPs and aptamer coating respectively (see Figure 4). Based on this discussion, by increasing the target cells’ concentration, the maximum current of apta-DS cannot be greater than the current related to bare GNPs. For this reason, as seen in this Figure 4a, when the concentration of cells is less than 25 cells/mL, the proposed apta-DS reveal a linear relationship. For higher cell concentrations (>25 cells/mL), the proposed apta-DS does not work linearly, however still it can be used as a detector verifying the presence of target cells.

## 4. Discussions

In this section, we discuss the advantages of the proposed apta-DS along with other practical considerations.

### 4.1. Optically Transparent apta-DS

In this paper we proposed a new optically transparent sensor for colon cancer detection by taking advantage of GNPs decorated on ITO. The GNPs are used as gold substrates for the immobilization of aptamer and trapping cancer cells. On the other hand, the tiny size of the aptamer does not significantly decrease the optical transparency. Therefore, as shown in Figure 7b, the trapped cells can be observed using low complexity optical microscope. The proposed apta-DS in this paper, in comparison with other reported techniques in literature [2,3,4,5,6,7,8] offers the advantage of exploiting both electrochemical and optical microscopic technique to verify the presence of cancer cells. In other words, the proposed apta-DS allows for the detection of cancer cells with high reliability. Therefore it is suitable for trapping, isolating and detecting the cancer cells required for research and disease diagnostic purposes.

### 4.2. Automated Blood Sample Preparation

As the main goal of this research is to detect cancer cells in blood samples, the required sample should be prepared from the whole blood. It is noteworthy that whole blood contains many different components including dissolved protein, antibodies, fats and other dissolved materials such as glucose and amino acids that may react with sensing layer on the top of electrode and consequently affect the functionality of sensors. In this project, a sample preparation procedure is used prior to introduce the sample to apta-DS. An alternative sample preparation solution is to use buffy coat extracted from whole blood sample. Buffy coat is a thin layer that can be distinguished and separated from plasma and red blood cells after centrifugation. Buffy coat only includes white blood cells and platelets. Therefore the presence of other cells such as circular tumor cells (CTCs) or other types of cancer cell can be accurately detected. Additionally, various automated biological instrumentation such as CELLSEARCH^®^ SYSTEM has been commercialized for detecting CTCs in the blood sample. This device, automatically detects the buffy-coat layer, allowing excess plasma to be aspirated to waste. In this paper, in a similar approach, as a long term objective, we aim to develop a novel automated system to separate buffy coat from blood sample and then it introduce the sample to the apta-DS. As the first step, in this paper, we successfully demonstrated the functionality of the proposed sensor for HCT 116 cell line using HEp-2 cell line as control. As the continuation of this project, in the future, we will develop an automated sample preparation system incorporated with the proposed apta-DS.

### 4.3. Improvement of the Limit of Detection

The design and implementation of apta-DS with lower limit of detection (LoD) is a key challenge in this field of study. For instance Cao et al. reported an aptasensor for tumor cell detection with a LOD equal to 40 cells/mL [14]. In another effort Wu et al. demonstrated and discussed the detection of 50 cells/mL using Au-ITO Hybrid Bipolar Electrode Amplification System [28]. Ahmadzadeh Raji et al. proposed an apta-DS with LOD equal to 7. As the continuation of that work [16], herein we proposed a new apta-DS for colon cancer detection with a LoD equal to 6 cells/mL.

In order to further improve the sensitivity of the proposed apta-DS, there are various potential solutions. For instance, a microfluidic structure can be used to direct the sample towards the aptamer. This structure likely increases the probability of trapping cancer cells by aptamers. On the other hand, an optical readout system can be incorporated with this apta-DS in order to detect the bonding between the aptamer of cells. As described in [29], GNPs deposited on ITO can be used as an alternative solution for high precision optical sensing purposes.

## 5. Conclusions

This paper described the development of an apta-DS for colon cancer detection using GNPs-decorated ITO. The capture aptamers were immobilized on the surface of GNPs through 11-MUA as a self-assemble monolayer for trapping the cancer cells expressing CEA. We demonstrated and discussed the experimental results using CV, FE-SEM and optical microscopic techniques. Based on these results, the proposed apta-DS exhibits a sensitivity of detecting 6 cells/mL. It is noteworthy that the proposed technique can also detect the epithelial cells (e.g., HP-29 and HCT116) expressing CEA, however in this paper we selected HCT116 to demonstrate this concept. The proposed apta-DS offers the advantages of easy-to-use, sensitive and reliable colon cancer detection suitable for colon cancer research and disease diagnostics. As a continuation of this work, we will develop a new platform consisting of microfluidic sample preparation device, and optical readout system for a fully automated high precision aptamer based colon cancer detection purposes.
